# Survival of 11,390 Continuum cups in primary total hip arthroplasty based on data from the Finnish Arthroplasty Register

**DOI:** 10.1080/17453674.2019.1603596

**Published:** 2019-04-17

**Authors:** Matias Hemmilä, Mikko Karvonen, Inari Laaksonen, Markus Matilainen, Antti Eskelinen, Jaason Haapakoski, Ari-Pekka Puhto, Jukka Kettunen, Mikko Manninen, Keijo T Mäkelä

**Affiliations:** a Department of Orthopaedic Surgery, University of Turku and Turku University Hospital, Turku;; b Department of Biostatistics, University of Turku, Turku;; c Coxa Hospital for Joint Replacement, Tampere;; d National Institute for Health and Welfare, Helsinki;; e Department of Orthopaedics and Traumatology, Oulu University Hospital, Oulu;; f Department of Orthopaedics and Traumatology, Kuopio University Hospital, Kuopio;; g Orton Hospital, Helsinki, Finland

## Abstract

Background and purpose — The use of trabecular metal (TM) cups for primary total hip arthroplasty (THA) is increasing. Some recent data suggest that the use of TM in primary THA might be associated with an increased risk of revision. We compared implant survival of Continuum acetabular cups with other commonly used uncemented cups.

Patients and methods — Data on 11,390 primary THAs with the Continuum cup and 30,372 THAs with other uncemented cups (reference group) were collected from the Finnish Arthroplasty Register. Kaplan–Meier survival estimates were calculated; the endpoint was revision for any reason, for infection, or for dislocation. Revision risks were assessed with adjusted Cox multiple regression models. A subgroup analysis on the use of neutral or elevated liners in the Continuum group was made.

Results — The 7-year survivorship of the Continuum group was 94.6% (95% CI 94.0–95.2) versus 95.6% (CI 95.3–95.8) in the reference group for revision for any reason. The risk for revision was higher in the Continuum group than in the reference group both for revision for any reason (HR 1.3 [CI 1.2–1.5)]) and for revision for dislocation (HR 1.9 [CI 1.5–2.3]). There was no difference in the rates of revision because of infection (HR 0.99 [CI 0.78–1.3]). Use of a neutral liner increased the risk for revision due to dislocation in comparison with the use of an elevated rim liner in the Continuum group (HR 1.7 [CI 1.2–2.5]).

Interpretation — THA with Continuum cups is associated with an increased risk of revision compared with other uncemented cups, mainly due to revisions because of dislocation. Our results support the use of an elevated liner when Continuum cups are used for primary THA.

Trabecular metal (TM) acetabular components were initially indicated in particular for cup revisions after total hip arthroplasty (THA) (Levine et al. 2006). TM cups provide increased bone ingrowth, better modulus of elasticity, and better stability due to their porous structure compared with conventional uncemented cup devices made of titanium alloy (Meneghini et al. 2010). Currently, TM revision cups are used frequently worldwide. Besides revision surgery, TM cups have demonstrated promising mid- to long-term survivorship in primary THA (Baad-Hansen et al. 2011, Howard et al. 2011) and hence the use of Continuum (ZimmerBiomet, Warsaw, IN, USA) TM cups in primary THA increases (Wegrzyn et al. 2015, De Martino et al. 2016). However, a recent register study showed that the early and mid-term revision rate of TM cups was slightly higher compared with other uncemented cups when used in primary THA in Sweden and Australia (Laaksonen et al. 2018).

The revision rate due to periprosthetic joint infection has been slightly increasing during recent decades (Dale et al. 2012). Some data suggest that the use of a TM acetabulum component in hip revision arthroplasty might be associated with a lower infection rate (Tokarski et al. 2015), but this finding has not been confirmed by register data and thus far there is, to our knowledge, no other evidence that the material of TM would protect patients from prosthetic joint infection (PJI) (Laaksonen et al. 2017, 2018).

It has also been suggested that there might be an increased risk of dislocations associated with the use of Continuum cups due to a decreased jumping distance of the femoral head (Pakarinen et al. 2018). To compensate for this circumstance, elevated or hooded acetabular liners are currently widely available for the purpose of decreasing the dislocation rate. The use of elevated liners may, in theory, improve hip stability and decrease the revision rate (Insull et al. 2014), but the routine use of elevated liners has been questioned (Krushell et al. 1991, Insull et al. 2014).

We compared the revision rate of Continuum cups used in primary THA with the most commonly used uncemented cups made of titanium alloy. We specifically compared the revision rates (1) for any reason, (2) for infection, and (3) for dislocation. We also studied whether use of elevated liners in primary THA decreases the revision rate due to dislocations compared with the Continuum cup with neutral liners.

## Patients and methods

This study is based on data from the Finnish Arthroplasty Register (FAR). The FAR data include nearly all hip and knee implants operated in Finland since 1980 (Paavolainen et al. 1991). Orthopedic units are obligated to provide all information essential for maintenance of the register to the Finnish National Institute for Health and Welfare. The register gathers information from most total hip implantations in the entire country and data coverage on primary THA exceeds 95% and on revision THA coverage is 81% (FAR 2018). Dates of death are obtained from the Population Information System maintained by the Population Register Centre. The data content of the register was scrutinized and revised in May 2014. The updated data now include detailed information on items like ASA class, BMI, and surgical approach.

### Study population

Between January 2009 and December 2017, 133,488 primary THAs were reported to FAR. In 11,390 of these the Continuum primary cup was used. The reference group consisted of the 6 most commonly used other uncemented cups made of titanium alloy (n = 30,372) (Table 1). A head size other than 28 mm, 32 mm, or 36 mm, dual mobility, and constrained liners were excluded. The number of patients with bilateral hip prostheses was 4,407 and in 658 patients both hips were operated simultaneously. 498 patients had the Continuum cup in one hip and a control group cup component in the contralateral hip. Tables 2 (see Supplementary data) and 3 show the demographic data hip-wise separately for the whole study period and after the data content revision in May 2014. Mortality during the study period in the Continuum group was 4% and 5% in the control group.

**Table 1.** Acetabular cups included in the study

Cup design	n (%)
Continuum (ZimmerBiomet, Warsaw, IN, USA)	11,390 (27)
Reference group	30,372 (73)
•Exceed (ZimmerBiomet, Warsaw, IN, USA)	1,550 (4)
•G7 (ZimmerBiomet, Warsaw, IN, USA)	1,121 (3)
•Pinnacle (DePuy, Warsaw, IN, USA)	14,844 (36)
•R3 (Smith & Nephew, Andover, MA, USA)	7,289 (18)
•Trident (shell) (Stryker, Mahwah, NJ, USA)	4,279 (10
•Vision Ringloc (ZimmerBiomet, Warsaw, IN, USA)	1,280 (3)

**Table 3.** Demographic data of the time period after data content revision in the Finnish Arthroplasty Register starting May 15, 2014. Values are frequency (%) unless stated otherwise

Data	Continuum group	Reference group
Mean age (SD)	67 (11)	66 (11)
BMI (SD)	28 (5)	28 (5)
Male sex	3,609 (42)	7,547 (46)
Diagnosis		
•Primary osteoarthritis	7,324 (85)	13,852 (85)
•Rheumatoid arthritis	137 (2)	195 (1)
•Other **^a^**	1,113 (13)	2,278 (14)
Femoral head size of prosthesis		
•28 mm	29 (0.3)	107 (1)
•32 mm	1,832 (21)	3,369 (21)
•36 mm	6,713 (78)	12,849 (79)
Status at end of follow-up		
•Not revised	8,202 (96)	15,792 (97)
•Revised	372 (4)	533 (3)
Liner material		
•Ceramic	619 (7)	2,249 (14)
•Highly cross-linked polyethylene	7,955 (93)	14,041 (86)
Elevated liner		
•No	4,385 (55)	8,648 (62)
•Yes	3,570 (45)	5,393 (38)
Approach		
•Posterior	6,654 (78)	12,884 (81)
•Anterolateral (modified Hardinge)	1,667 (20)	2,864 (18)
•Anterior (Watson-Jones)	15 (0.2)	11 (0.1)
•Anterior (Smith-Peterson)	143 (2)	137 (1)
Trochanteric osteotomy performed	1 (0.01)	1 (0.01)
ASA class		
•1	1,281 (15)	2,163 (14)
•2	4,132 (49)	8,260 (52)
•3	2,992 (35)	5,308 (33)
•4	104 (1)	189 (1)
Femoral stem fixation		
•Uncemented	5,502 (65)	13,209 (81)
•Cemented	3,030 (36)	3,057 (19)

a Fractures (5% Continuum group vs. 4% control group), avascular necrosis (3% vs. 3%), osteoarthritis due to hip dysplasia (2% vs. 2%), tumors (1% vs. 1%), congenital hip dislocation (0.5% vs. 0.3%), inflammatory arthritis (0.3% vs. 0.4%), Legg–Perthes–Calve disease (0.3% vs. 0.2%), femoral head epiphyseolysis (0.2% vs. 0.1%), status post purulent arthritis (0.1% vs. 0%).

**Table 4.** Indication for revision prior to data content revision (May 15, 2014) of the Finnish Register. Values are frequency (%)

Main reason for revision^a^	Continuum group	Reference group
Aseptic loosening		
•Cup and stem	0 (0)	2 (1)
•Cup	2 (4)	15 (5)
•Stem	3 (6)	17 (5)
Infection	7 (13)	56 (18)
Dislocation	21 (39)	88 (28)
Component malposition	3 (6)	31 (10)
Fracture	13 (24)	72 (23)
Component breakage	0 (	4 (1)
Other	5 (9)	28 (9)

a No data available concerning indication for revision from 83 revisions.

### Surgery

In the Continuum group, 36 mm femoral heads were used in 79% of cases. The corresponding proportion in the reference group was 80%. A ceramic liner was used in 14% of cases in the Continuum group and in 27% of cases in the reference group. The rest were highly cross-linked polyethylene liners in both groups. From May 2014 surgical approach data have been available from the register. Since then the majority of the operations have been performed via the posterior approach in both groups (79% in the Continuum group and 81% in the reference group). Uncemented femoral stems were used in 71% in the Continuum group compared with 83% in the reference group. The average follow-up time was 3 years (0–9) in the TM group and 4 years (0–10) in the reference group.

### Statistics

Kaplan–Meier survival estimates were calculated for both groups and the log rank test was used to compare the survival curves. Revision was described as change or removal of at least 1 component (Tables 4 and 5). To reduce the risk of selection bias we adjusted the estimated revision risks in the Cox multiple regression model by sex, age group, diagnosis, femoral head size, operated side, operation year, and fixation of the femoral stem. An additional cup revision analysis was performed and the type of approach, ASA, BMI, and elevation status of the liner were added to the Cox model as possible confounders for cup revision for any reason as the endpoint. The analysis was done with the data of primary operation after register update in May 2014. In the Continuum elevation subgroup analysis sex, age group, diagnosis, side, stem fixation, and operation year were added to the Cox model (head size was stratified) and other than polyethylene liners were excluded. If the proportional hazards assumption for a variable was not fulfilled in the Cox model, the model was stratified by it instead. Stratification in Cox models means that the hazard functions can be estimated for all level combinations of the stratified variables, and the hazard ratios for the other variables (those that meet the proportional hazard assumption) are then optimized for all these hazard functions. Without stratification we would assume that hazards were the same for all levels of such variables.

**Table 5.** Indication for revision after new indications for revision were added at the data content revision (May 15, 2014) of the Finnish Register. Values are frequency (%)

Main reason for revision^a^	Continuum group	Reference group
Aseptic loosening		
•Cup	5 (1)	10 (2)
•Stem	15 (4)	26 (4)
Osteolysis		
•Cup	2 (1)	8 (1)
•Stem	1 (0.3)	11 (2)
Liner wear	0 (0)	2 (2)
Component breakage		
•Cup	0 (0)	1 (0.2)
•Liner	1 (0.3)	11 (2)
•Head	1 (0.3)	1 (0.2)
•Modular neck	0 (0)	1 (0.2)
Infection	100 (26)	194 (30)
Dislocation	132 (34)	153 (24)
Component malposition		
•Cup	12 (3)	23 (4)
•Stem	1 (0.3)	14 (2)
Periprosthetic fracture		
•Acetabulum	6 (2)	2 (0.3)
•Femur	73 (19)	105 (17)
Adverse reaction to metal debris	2 (1)	5 (1)
Squeaking	2 (1)	5 (1)
Unexplained pain	10 (3)	32 (5)
Leg length discrepancy repair	4 (1)	10 (2)
Other	17 (4)	24 (4)

a No data available concerning indication for revision from 83 revisions.

The primary outcome was revision for any reason and the secondary outcomes were revision for periprosthetic infection, revision for dislocation, and cup revision for any reason. Patients were censored for any event other than the outcome, or at the end of the follow-up. After the register update in May 2014 it has been possible to assess separately which component has been changed or removed in connection with the revision. Therefore, a subgroup analysis for cup-only-revisions was performed only for the newest FAR data. In addition, a subgroup analysis was performed for Continuum cups by liner type (neutral or elevated liner) with dislocation revision as the endpoint. Survival data are presented as percentages with the 95% confidence interval (CI). Cox regression analysis is presented with the hazard ratio (HR) and the CI.

All analyses were performed using the SAS software (Version 9.3; SAS Institute, Cary, IN, USA).

### Ethics, funding, and potential conflicts of interest

Ethical approval: June 13, 2017, Dnor THL/926/5.05.00/2017. This research received no funding. The authors declare no conflicts of interest.

## Results

### 

#### Revision for any reason

**Figure 1.**
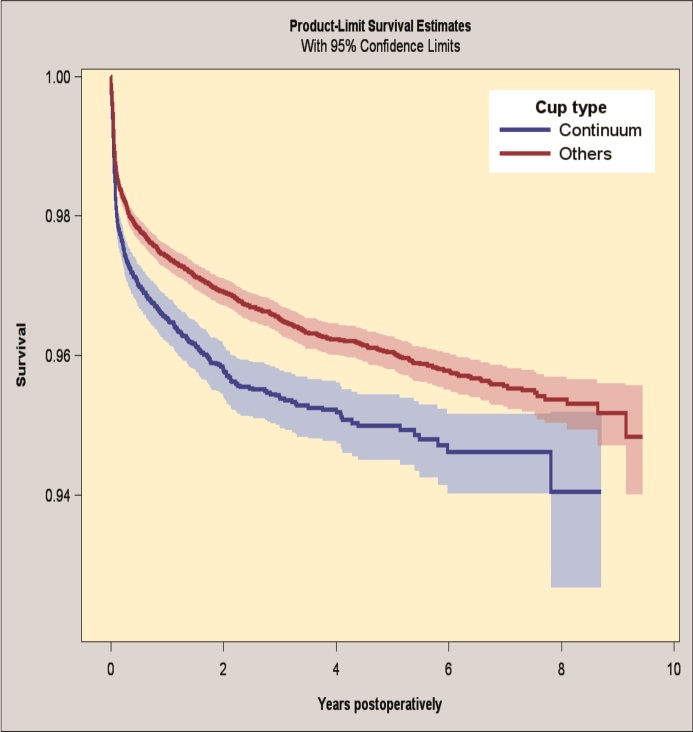
Kaplan–Meier survival for Continuum group and reference group with revision for any reason as the endpoint. 95% CI levels presented in blue and red.

The up to 7-year survivorship for the Continuum group was 94.6% (CI 94.0–95.2) and the survival for the reference group was 95.6% (CI 95.3–95.8) for revision for any reason as an endpoint (Figure 1, Table 6; see Supplementary data). By Cox regression analysis the Continuum group had an increased risk of revision for any reason compared with the reference group (HR 1.3, CI 1.2–1.5).

#### Cup revision for any reason

In the cup-only-revision analysis performed with the data from May 15, 2014 to December 31, 2017, the 3-year survivorship was the same in the Continuum group as in the reference group: 99.4% vs. 99.6% (CI 99.2–99.6 vs. 99.5–99.7). These figures are not statistically different (Cox regression analysis HR 1.3, Cl 0.8–2.0).

#### Revision due to infection

**Figure 2.**
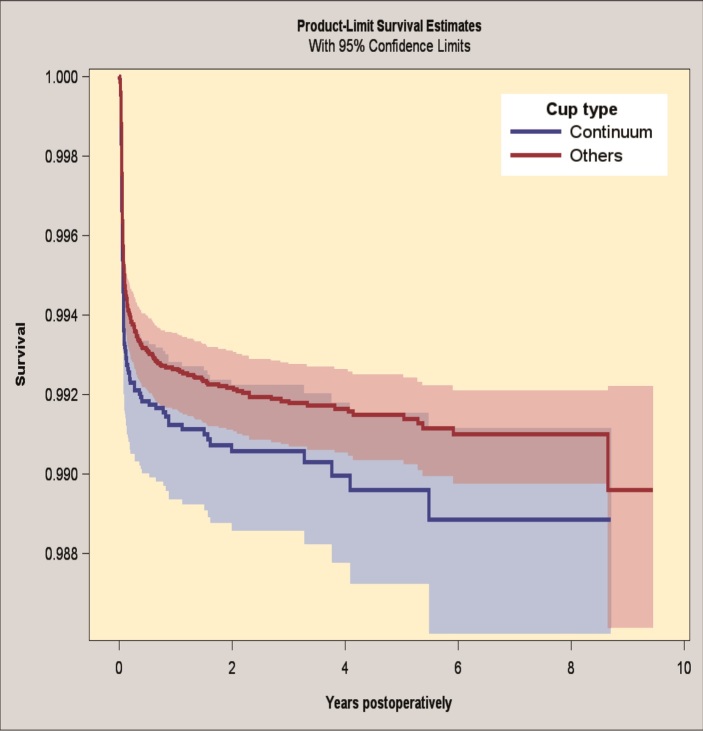
Kaplan–Meier survival for Continuum group and reference group with revision for infection as endpoint. 95% CI levels presented in blue and red.

The 7-year survivorship for the Continuum group was 98.9% (CI 98.6–99.1) and for the reference group 99.1% (CI 99.0–99.2), when revision because of infection was the endpoint (Figure 2). The risk of revision for infection was the same in the groups (HR 1.0, CI 0.8–1.3) (Table 7; see Supplementary data).

#### Revision due to dislocation

**Figure 3.**
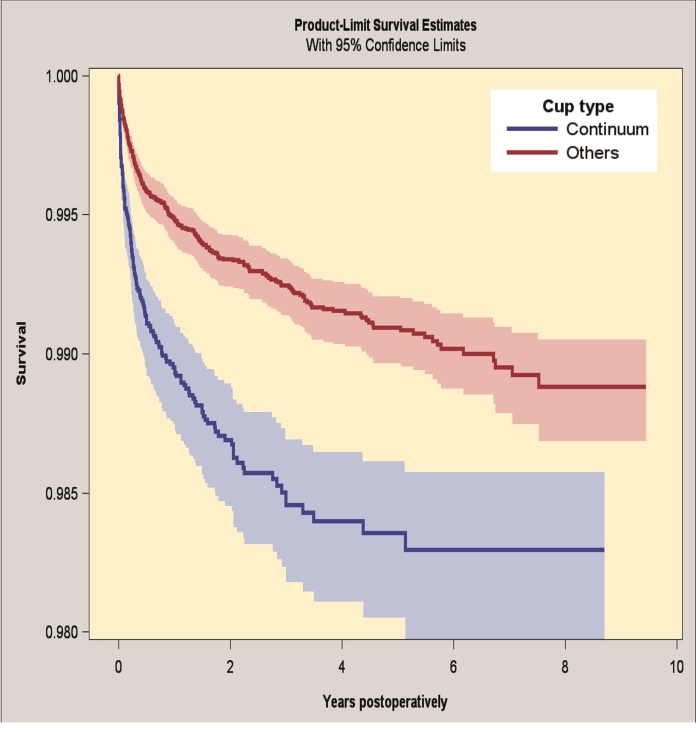
Kaplan–Meier survival for Continuum group and reference group with revision for dislocation as endpoint. 95% CI levels presented in blue and red.

The 7-year survivorship for the Continuum group was 98.3% (CI 98.0–98.6) and for the reference group 99.0% (CI 98.8–99.1), when revision because of dislocation was the endpoint (Figure 3). The Continuum group had an increased risk of revision for dislocation (HR 1.9, CI 1.5–2.3) compared with the reference group (Table 7; see Supplementary data).

#### Subgroup analysis: Continuum THA with or without liner elevation

**Figure 4.**
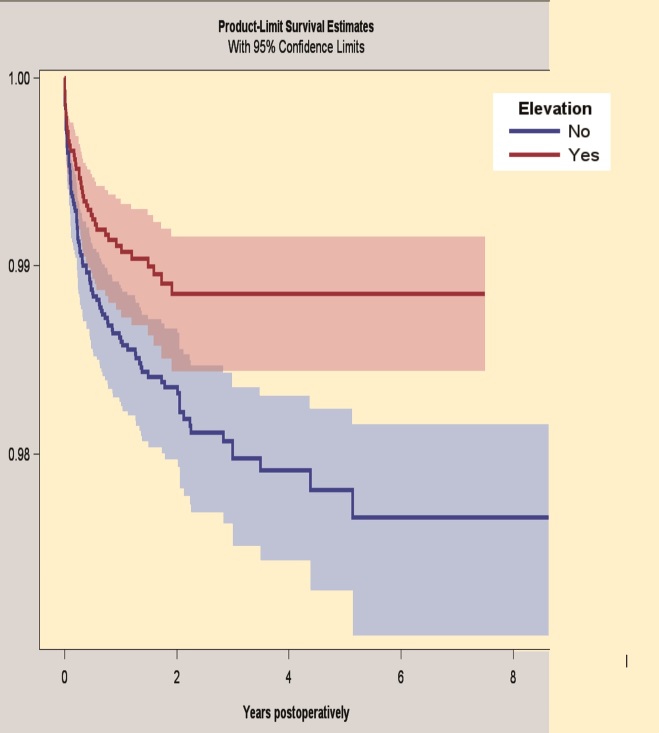
Kaplan–Meier survival by subgroup analysis of Continuum THA with or without elevated liner. Endpoint: revision for dislocations. 95% CI levels presented in blue and red.

The 5-year survivorship for the Continuum group with elevated liners was 98.9% (CI 98.4–99.2) and for the Continuum group with neutral liners 97.8% (CI 97.3–98.2), when revision because of dislocation was the endpoint (Figure 4). After adjustments of the statistical data, the Continuum group with neutral liners had a higher risk of revision for dislocation compared with the Continuum group with elevated liners (HR 1.7, CI 1.2–2.5).

## Discussion

This study shows that use of the Continuum THA is associated with a slightly higher risk of revision than use of other uncemented titanium alloy cups. The Continuum study group and the reference group had a similar risk of revision due to infection, but the risk of revision due to dislocation was higher in the Continuum group. Further, the use of elevated liners in the Continuum THA reduced the risk of revision for dislocation compared with neutral liners.

Trabecular metal was first introduced to the market in 1997. Since then, TM cups have shown reliable results when used for hip revision arthroplasty and are currently used routinely worldwide (Davies et al. 2011, Mohaddes et al. 2015). Their routine use in primary THA is increasing. Implant survival of primary TM cups has been comparable or even superior compared with uncemented devices made of titanium alloy (Baad-Hansen et al. 2011, Howard et al. 2011, Wegrzyn et al. 2015, De Martino et al. 2016). However, a recent collaborative register study reported that TM cups have a 1.5 times higher risk for revision than other frequently used uncemented cups in primary THA (Laaksonen et al. 2018). These results were somewhat surprising and at variance with previous literature. Our study supports the previous finding from the Swedish and Australian registries of a higher risk of revision of TM cups.

The use of TM cups in primary THA is increasing in Sweden and Australia (Laaksonen et al. 2018). Continuum was the 2nd most common cup design in the FAR data of the present study. Due to the good gription of and high primary stability of TM, Continuum cups have been preferred in more demanding THAs. To reduce the risk of selection bias towards more difficult cases being treated with Continuum cups, we adjusted the revision risks in the Cox regression models. Our data suggest that the use of the Continuum cup in primary THA does not give superior results compared with other uncemented devices. However, TM cups are a reliable option when treating large bone defects in revision or complex primary THA and results in these cases have been excellent (Weeden and Schmidt 2007, Macheras et al. 2010). The revisions in the Continuum group in the current study were mainly due to dislocations, and the number of revisions for early lack of osteointegration or aseptic loosening was low.

PJIs are a growing challenge as an increasing number of joint replacements are being performed and the life expectancy of patients is increasing (Huotari et al. 2015). Indeed, the cumulative incidence of PJI in USA and the Nordic countries is reportedly growing (Dale et al. 2012, Kurtz et al. 2012). A recent study presented promising results of TM components possibly having a protective effect against PJI (Tokarski et al. 2015). These results were not confirmed in a register study (Laaksonen et al. 2018), and were similar to our results: the risk for revision due to PJI was similar in the Continuum and in the reference group (Table 7; see Supplementary data).

Continuum cups with the neutral liner used have been associated with a reduced jumping distance of the femoral head and possibly with a higher dislocation risk due to this circumstance (Pakarinen et al. 2018). In an earlier large register study based on Australian and Swedish data, the revision risk due to dislocation was not assessed separately, although the overall revision risk of TM cups was increased compared with the other uncemented cups (Laaksonen et al. 2018). We found that the risk of revision due to dislocation of the Continuum THA is increased compared with reference THAs. This difference is largely explained by the difference in the revision rate due to dislocation. In the subgroup analysis of the Continuum group we found that cups with a neutral polyethylene liner are associated with 1.7-fold dislocation revision risk compared with Continuum cups with an elevated liner. This is in line with the previous finding by Pakarinen et al. (2018).

Elevated liners were first introduced by Charnley in the early 1970s to decrease the tendency for posterior dislocation by providing more coverage (Charnley 1979). The improved stability in primary THA while using an elevated rim liner was first reported in 1996 and, although these liners are widely used, there is only limited clinical evidence to support their use (Cobb et al. 1996, Sultan et al. 2002, Carter et al. 2011). Also, the benefit of routine use of elevated-rim liners in instances in which the acetabular component otherwise is positioned satisfactorily has been questioned (Krushell et al. 1991). In addition, there might be potentially harmful side effects. The elevated liners may predispose the neck of the prosthesis to impinge on the acetabular rim, forcing the head out of the cup anteriorly, but such a risk has not been confirmed in clinical studies (McCollum and Gray 1990, Sultan et al. 2002). Despite these suspicions, elevated liners have not been associated with increased revision rates during 5 years of follow-up (Cobb et al. 1997). Also, the use of lipped liners with modular uncemented acetabular components has been associated with a decreased rate of revision due to instability after primary THA, according to a register study from New Zealand (Insull et al. 2014). Our data support these findings: we did not observe any trend toward an elevated risk of revision due to increased wear. It is nevertheless prudent to remember that these problems may appear in a longer follow-up.

Our study has some limitations. First, we were not able to assess radiographs to evaluate preoperative bone loss. It is possible that Continuum cups have been used in more demanding cases. However, Continuum being the second most used uncemented cup during our study time does suggest that it is used routinely for primary THA. Second, we were able to analyze only factors included in the register dataset. It is possible that patients might have comorbidities that could influence their dislocation risk that we are not aware of. Third, we were only able to use revision as the outcome. Some of the patients might have experienced pain, dislocations, or other implant-related problems without having a revision, for example, due to poor general health contraindicating risky revision surgery.

In summary, this large nationwide study shows that the use of the Continuum cup for primary THA does not provide an advantage over traditional uncemented cups. On the contrary, the use of Continuum cups was associated with an increased revision risk compared with other uncemented cups. This enhanced risk was largely due to revisions for dislocations. If the Continuum cup is used, our results support the use of the elevated rim liner, rather than the neutral rim liner, as the primary choice.

## Supplementary Material

Supplemental Material
