# Supplementary material for: Survival of 11,390 Continuum cups in primary total hip arthroplasty based on data from the Finnish Arthroplasty Register
Source: Acta Orthop. 2019 Apr 17;90(4):312–7. doi: 10.1080/17453674.2019.1603596 (PMC6718180; doi:10.1080/17453674.2019.1603596)
Supplement: Supplemental Material [file IORT_A_1603596_SM9380.pdf]

Supplementary data

Table 2. Demographic data for the whole study period. Values are frequency (%) unless stated otherwise

Data	Continuum group	Reference group
Mean age (SD)	66 (11)	66 (11)
BMI (SD)	28 (5)	28 (5)
Male	4,703 (41)	13,604 (45)
Diagnosis		
Primary osteoarthritis	9,789 (86)	26,152 (86)
Rheumatoid arthritis	210 (2)	505 (2)
Other	1,391 (12)	3,715 (12)
Femoral head size of prosthesis		
28 mm	34 (0.3)	361 (1)
32 mm	2,362 (21)	5,868 (19)
36 mm	8,994 (79)	24,143 (80)
Status at end of follow-up		
Not revised	10,900 (96)	29,272 (96)
Revised	490 (4)	1,100 (4)
Liner material		
Ceramic	1,633 (14)	8,150 (27)
Highly cross-linked polyethylene	9,757 (86)	22,044 (73)
Elevated liner		
No	5,589 (57)	12,404 (56)
Yes	4,168 (43)	9,640 (44)
Operation year		
2009–2013	2,370 (21)	12,576 (41)
2014–2017	9,020 (79)	17,796 (59)
Femoral stem fixation		
Uncemented	7,988 (71)	24,877 (83)
Cemented	3,234 (29)	4993 (17)

Table 9. Risk of cup revision for any reason from May 15, 2014 by Cox regression model (adjusted for age group, diagnosis, head size, side, ASA, BMI, stem fixation, liner elevation, approach and stratified by sex)

Group	HR (95% CI)
Continuum vs. others	1.31 (0.84–2.04)
18–55 vs. 76+	0.58 (0.22–1.52)
56–65 vs. 76+	0.79 (0.40–1.53)
66–75 vs. 76+	0.78 (0.45–1.36)
Other vs. rheumatoid arthritis	0.49 (0.16–1.49
Primary osteoarthritis vs. rheumatoid arthritis	0.28 (0.10–0.78)
Head size 28 mm vs. 36 mm	5.29 (0.71–39.4)
Head size 32 mm vs. 36 mm	2.95 (1.81–4.80)
Left vs. right	1.11 (0.72–1.70)
ASA, 1 level increase	1.26 (0.88–1.80)
BMI, 1 unit increase	0.99 (0.95–1.04)
Cemented vs. uncemented	0.87 (0.51–1.47)
Elevated liner, no vs. yes	1.72 (1.06–2.82)
Approach	
Posterior vs. anterior	0.75 (0.18–3.09)
Anterolateral vs. anterior	0.33 (0.07–1.52)

Table 6. Kaplan–Meier survivals (%) for Continuum group and the reference group in primary THA with revision for any reason as the endpoint at different time points

Implant type	n	Revisions (n)	At risk	Up to 1 year survival (CI)	At risk	Up to 3 years survival (CI)	At risk	Up to 5 years survival (CI)	At risk	Up to 7 years survival (CI)
Continuum group	11,390	490	9,300	96.5 (96.2–96.9)	4,160	95.4 (95.0–95.8)	1,741	95.0 (94.5–95.4)	462	94.6 (94.0–95.2)
Reference group	30,372	1,100	27,249	97.4 (97.2–97.6)	17,836	96.5 (96.3–96.7)	9,777	96.0 (95.8–96.3)	3,575	95.6 (95.3–95.8)

Table 7. Revision risk according to Cox regression model (adjusted for age group, gender, diagnosis, femoral head size, operated side, operation year group, and fixation of the femoral stem) revision for any reason, revision for infection, revision for dislocation, any cup revision as the endpoints

	Reference group (Ref.)	Continuum group HR (95% CI)
Revision for any reason	1.0	1.3 (1.2–1.5)
Revision for infection	1.0	0.99 (0.8–1.3)
Revision for dislocation	1.0	1.9 (1.5–2.3)
Cup revision as the endpoint	1.0	1.3 (0.8–2.0)

Table 8. Risk of revision for any reason during the whole time period by Cox regression model (adjusted for side, stem fixation, age group, diagnosis, head size and stratified by sex, operation year)

Group	HR (95% CI)
Continuum vs. others	1.30 (1.17–1.46)
Left vs. right	1.00 (0.90–1.10)
Cemented vs. uncemented	0.81 (0.71–0.94)
18–55 vs. 76+	0.64 (0.53–0.78)
56–65 vs. 76+	0.77 (0.67–0.90)
66–75 vs. 76+	0.80 (0.70–0.92)
Other vs. rheumatoid arthritis	0.98 (0.70–1.39)
Primary osteoarthritis vs. rheumatoid arthritis	0.65 (0.47–0.90)
Head size 28 mm vs. 36 mm	1.23 (0.77–1.96)
Head size 32 mm vs. 36 mm	1.05 (0.92–1.20)
